# Item distribution, scalability and internal consistency of the QUALIDEM quality of life assessment for patients with dementia in acute hospital settings

**DOI:** 10.1186/s12955-023-02094-1

**Published:** 2023-01-31

**Authors:** Daniel Lüdecke, Martin Nikolaus Dichter, Stefan Nickel, Christopher Kofahl

**Affiliations:** 1 Institute of Medical Sociology, University Medical Center Hamburg-Eppendorf, Martinistraße 52, 20246 Hamburg, Germany; 2 Institute of Nursing Science, University of Cologne Medical Faculty and University Hospital Cologne, University of Cologne, Cologne, Germany

**Keywords:** QUALIDEM, Quality of life, Patients with dementia, Hospitals, Validation

## Abstract

**Background:**

Quality of life (QoL) of people with dementia (PwD) is an important indicator of quality of care. Studying the impact of acute hospital settings on PwD’s QoL requires assessment instruments that consider environmental factors. Until now, dementia-specific QoL instruments have not yet demonstrated their feasibility in acute hospitals because their use takes up too much time or their validity depends on observation periods that usually exceed the average length of hospital stays. Therefore, validated instruments to study QoL-outcomes of patients with dementia in hospitals are needed.

**Methods:**

Data stem from a study that analyzed the impact of a special care concept on the QoL of patients with dementia in acute hospitals. Total sample size consisted of N = 526 patients. Study nurses were trained in using an assessment questionnaire and conducted the data collection from June 2016 to July 2017. QoL was assessed with the QUALIDEM. This instrument consists of nine subscales that can be applied to people with mild to severe dementia (N = 344), while six of the nine subscales are applicable for people with very severe dementia (N = 182). Scalability and internal consistency were tested with Mokken scale analysis.

**Results:**

For people with mild to severe dementia, seven out of nine subscales were scalable (0.31 ≤ H ≤ 0.75). Five of these seven subscales were also internally consistent (ρ ≥ 0.69), while two had insufficient reliability scores (ρ = 0.53 and 0.52). The remaining two (*positive self-image*, *feeling at home*) subscales had rather low scalability (H = 0.17/0.16) and reliability scores (ρ = 0.35/0.36). For people with very severe dementia, all six subscales were scalable (0.34 ≤ H ≤ 0.71). Five out of six showed acceptable internal consistency (ρ = 0.65–0.91). Only the item s*ocial relations* had insufficient reliability (ρ = 0.55).

**Conclusions:**

In comparison with a previous evaluation of the QUALIDEM in a long-term care setting, the application in a hospital setting leads to very similar, acceptable results for people with mild to severe dementia. For people with very severe dementia, the QUALIDEM seems to fit even better in a hospital context. Results suggest either a revision of unsatisfactory items or a general reduction to six items for the QUALIDEM, for all PwD. In general, the QUALIDEM can be recommended as instrument to assess the QoL for PwD in the context of hospital research. Additionally, an investigation of the inter-rater reliability is necessary because the qualification of the nurses and the length of stay of the patients in the hospital differ from the previous investigations of the inter-rater reliability of QUALIDEM in the nursing home.

**Supplementary Information:**

The online version contains supplementary material available at 10.1186/s12955-023-02094-1.

## Background

Acute hospitals face the challenge of changes in demographic and clinical characteristics of people who need acute health care, which leads to an increased prevalence of people with dementia (PwD) [1, 2]. According to current studies and systematic reviews, there are no precise numbers on the prevalence of cognitive impairment in patients in hospitals. Most studies, however, indicate that approximately 40% of inpatients have at least mild cognitive impairments or are diagnosed with dementia [3].

Many hospitals and their personnel are insufficiently prepared for those people with cognitive impairments, especially in acute care units predominantly focusing on somatic diseases [4]. This results in an increased likelihood of complications during the hospital stay and post-operative complications, which in turn affect the quality of life (QoL) of PwD [5–7]. However, QoL is an important indicator of quality of care and a major dimension when assessing patient reported outcomes. This particularly holds true for older people, regarding global outcome measures for interventions [8, 9].

Therefore, psychometrically validated instruments to measure QoL of PwD in hospital contexts are strongly needed. A recent systematic review and meta-regression analysis by Li et al. reveals a number of generic instruments such as the EuroQol five-dimension questionnaire (EQ-5D) and dementia-specific instruments such as the DEMQOL-U [10]. Most instruments, however, are not feasible to assess QoL in acute hospitals. Usually, QoL instruments for PwD are only validated in nursing home care settings. The use of instruments developed for a nursing home care settings take too long when used in hospitals. Their validity depends on observation periods that usually exceed the average length of a hospital stay. Additionally, the critical life-event of hospitalization has a direct impact on QoL. Another issue is the qualifications and experience of nurses in caring for people with dementia, which differs between hospitals and nursing homes. This might be relevant for a proxy instrument. Therefore, previous studies on psychometric properties of QoL instruments are not directly transferable to a hospital setting.

This also applies to the recently developed QUALIDEM instrument, too [11, 12]. QUALIDEM is based on the adaptation-coping model [13] and defines dementia-specific QoL as a multidimensional assessment of the individual person-environment system in terms of adaptation to the perceived consequences of dementia [11]. This means that the dementia-specific QoL is the result of a successful or unsuccessful adaptation of the PwD to the physical, psychological and social consequences of the dementia syndrome.

Against this background, the aim of this paper is to investigate whether the item distribution, scalability and internal consistency of the subscales of the German version of the QUALIDEM instrument can be replicated in a hospital context, to draw conclusions about the applicability of the QUALIDEM in hospital research regarding PwD. However, proxy ratings with an instrument as QUALIDEM are accompanied by methodological challenges, and the results are systematically lower than those for self-rated QoL [14].

## Methods

### Design

Primary data was collected in a study called “DAVID” (German acronym for Diagnostics, Acute therapy, Validation at an Internal medicine ward for patients with Dementia) that compared the quality of care for patients with dementia within an internal medicine unit using a specialized dementia care concept as opposed to regular care in acute hospitals. The study was designed as a cross-sectional study, including two internal medicine wards in two hospitals located in Hamburg, Germany [15].

Prior to the study, a study protocol was developed and submitted to the ethical committee of the medical association of Hamburg. The ethical committee approved the proposal and confirmed that the study conforms to ethical and legal requirements (approval code PV5102). Study participants were not able to give their informed consent due to their cognitive impairments. However, as data mostly derived from the hospitals’ regular documentation, and as data was completely anonymous, the ethics committee waived the need of an informed consent.

### First sample site

The special care ward “DAVID” was an internal medicine ward in the Protestant Hospital Alsterdorf, a not-for-profit organization, and had 14 beds. During the 12 months of data collection, 349 patients were treated. The ward employed nine care workers as nursing staff. Key components of the special care concept were a specific architectonical design, including a homelike lounge or a specific coloring of doors and walls; doctors, nurses and service staff were trained in coping with challenging behavior and other dementia related issues, e.g. using basal stimulation or validation therapy; mobile devices for diagnostics, to perform as many treatments as possible in the different rooms of the special care ward; involvement of relatives regarding assessment, care and discharge planning; and regular therapeutic offers like occupational or speech therapy, plus social offers like music, playing games or nurses spending more time than usual to care for the patients.

### Second sample site

The regular care ward was part of a larger private-company hospital with emergency hospitalization. It had 80 beds and during the 12 months of data collection, about 3500 patients were treated in this internal medicine ward. Twenty-six employees worked as care staff in this ward. Trainees supported the care team. The regular care ward had no specific care concept for dementia patients. The care staff was not particularly trained in dementia topics.

### Data collection and participants

An assessment questionnaire was developed to obtain data from PwD. Study nurses were trained in using this assessment questionnaire and then conducted the data collection in both hospitals. The assessment questionnaire comprised items on different domains like QoL, functional limitations, cognitive status, comorbidities, agitation or challenging behavior. Participants were observed for about 1 week (depending on the length of stay). The study nurses then rated the participants’ outcomes for these domains. Two study nurses were responsible for data collection in the special care ward and one study nurse for the data collection in the regular care ward. Data was collected from June 2016 to July 2017. People with dementia were included when they showed at least mild cognitive impairments or memory problems. A short dementia screening using the Salzburg dementia test prediction (SDTP) [16] was carried out by the study nurse to assess the severity of dementia of patients who had no clarified dementia diagnosis, and to identify further patients who would qualify for the study. Patients were excluded when they were not responsive or completely confined to bed due to severe health-related dependency. As both care wards had no particular selection criteria for patients such as age, mobility, or the main diagnosis that lead to hospital admission, no further exclusion criteria for the study were defined. The total sample size for the present analysis consists of N = 526 people with dementia (special care ward: n = 333; regular care ward: n = 193).

### Measurements

For the description of the sample, information on age, gender, length of stay, functional limitations, challenging behavior, comorbidities and quality of life were used. Functional limitations in daily living were assessed with the Barthel-Index [17]. This score ranged from 0 (completely dependent) to 100 points (no basic functional limitations). Agitation and challenging behavior of patients was assessed using the Pittsburgh Agitation Scale (PAS) [18] ranging from 0 to 16 points (higher scores indicate stronger agitation). A modified version of the Charlson’s Comorbidity Index (CCI) was built to represent comorbidities and chronical diseases [19].

The QUALIDEM (Version 1) [11, 12] was used to assess the QoL of PwD. QUALIDEM for people with mild to severe dementia comprises 37 items reflecting nine different subdomains of QoL: “care relationship” (7 items, 0–21 points), “positive affect” (6 items, 0–18 points), “negative affect” (3 items, 0–9 points), “restless and tense behavior” (3 items, 0–9 points), “positive self-image” (3 items, 0–9 points), “social relations” (6 items, 0–18 points), “social isolation” (3 items, 0–9 points), “feeling at home” (4 items, 0–12 points) and “have something to do” (2 items, 0–6 points). For individuals with very severe dementia, only six of the nine subscales apply (with a total of 18 items), hence the dimensions “positive self-image”, “feeling at home” and “have something to do” were omitted. For each subscale, higher values indicate higher QoL. In the QUALIDEM questionnaire, not all of the 37 items were coded in the same direction. The reason is that for some items higher values mean a better QoL, while other items were coded so that lower values indicate better QoL. Thus, where necessary, items were recoded so higher values always indicate higher QoL. In the original version of the QUALIDEM, which was developed for long-term care settings, some items used the wording “residents”. In the present study, the term “patients” was used, which is more appropriate in a hospital setting.

The Mini Mental Status Examination test [20] was used to assess the severity of dementia. The score ranges from zero (very strong cognitive impairments) to 30 (very mild or no cognitive impairments) points. A cut-off score of MMSE < 10 indicates very severe dementia in patients.

### Statistical analysis

#### Sample description

The descriptions of the participants, the missing data, and the item distributions were based on descriptive statistics. Statistically significant differences of *p* < 0.05 between the two groups of “mild to severe” and “very severe” dementia were tested using t-tests, χ^2^-tests or Mann–Whitney-U-tests, depending on the level of measurement and distribution of variables. Since the QUALIDEM subscales differed in the number of items contributing to each subscale, we normalized the subscale scores (for the figures only), so each subscale in the figures ranged from 0 to 1. This allowed a more intuitive comparison of QUALIDEM subscales because they no longer had different ranges.

#### Item distribution and floor/ceiling effects

The item distribution for all QUALIDEM items was reported and the difficulty for each item was calculated to indicate floor (item difficulty < 0.2) or ceiling (item difficulty > 0.8) effects per item, which means items had poor discrimination if these thresholds were exceeded [21]. Furthermore, floor and ceiling effects for subscale scores and the QUALIDEM total score were determined by calculating the proportions of PwD appearing in the lower or upper 10% of each score [22]. Floor or ceiling effects larger than 15% were considered as statistically significant and indicated poor discrimination of a scale [23].

#### Known-group validity

To assess how well the QUALIDEM distinguishes among distinct groups, we calculated the known-group validity [24]. Distinct groups were build based on five different characteristics: age, sex, functional limitations (Barthel-Index), agitation and challenging behavior (PAS-score) and morbidity (CCI). Therefore, all continuous characteristics were dichotomized at the median. For each characteristic, hypotheses were defined a priori. Prior assumptions were based on research on this topic [25, 26]:

1. QoL is not significantly associated with age, hence we expect no significant differences in QoL by age, because our selection of the sample only contains older aged patients.
2. QoL is not significantly associated with gender. We expect no significant differences between male and female patients.
3. QoL is negatively associated with functional limitations. We expect lower QoL scores for higher functional limitations.
4. We expect significantly lower QoL when PwD show higher agitation and challenging behavior.
5. QoL is negatively associated with morbidity. The higher the number of comorbidities, the lower the QoL scores.

Differences among groups were tested for statistical significance using one-sided or two-sided t-tests. Cohen’s d was used to indicate the effect size. A coefficient < 0.2 was considered as very small, 0.2 to < 0.5 as small, 0.5 to < 0.8 as medium and 0.8 and higher as large effect [27].

#### Scalability and internal consistency

Scalability and internal consistency of the QUALIDEM subscales were analyzed with the confirmatory Mokken scale analysis (MSA) [28–30], which is a scaling procedure for both dichotomous and ordinal polytomous items. It assesses whether a number of items measure the same underlying concept of a scale. MSA has been widely used in QoL research and is the preferred method for instruments like the QUALIDEM that consist of ordinal data [12, 31, 32]. The scalability of scales was measured by Loevinger's coefficient H, in short just “H”. It indicates the internal correlation of each subscale. Mokken [28] proposed the following rules of thumb for this coefficient: A scale was considered weak if 0.3 ≤ H < 0.4, moderate if 0.4 ≤ H < 0.5, and strong if H ≥ 0.5. If H was lower than 0.3, an item or scale was considered “not scalable”, which means items were unrelated, thus not reflecting the underlying concept of a scale. The correlation between a single item and the remaining items of a scale was expressed by the value “H_i_”, which should be non-negative to fulfil the assumptions of the MSA, and should be higher than 0.3 to show at least moderate discrimination power, thereby being useful for the scale [24]. The criterion of the MSA (“crit”, [33]) was used to check monotonicity assumptions. This assumption relates to the probability of a particular item level or the correct answer is a monotonically non-decreasing function of the latent trait of that item [34].

Finally, the Molenaar Sijtsma statistic (“rho”, ρ) as well as Cronbach’s α were calculated as reliability measures for the internal consistency of scales [35, 36], the latter mainly for comparison to other study results. For both ρ and α, a value smaller than 0.6 indicated insufficient internal consistency of a scale, while values above 0.7 were acceptable or satisfying. Scales with ρ or α between 0.6 and 0.7 were sufficient, but questionable.

For the present MSA, missing values were imputed using the suggested two-way imputation [37, 38]. In a second step, missing data were imputed using the multivariate imputation by chained equations method [39], in order to compare how different imputation methods affect the results of the MSA (these results are shown in the Additional file 1: Table A1).

All analyzes were performed using the R statistical package [40] with the R packages *mokken* [41], *mice* [39], *effectsize* [42] and *sjPlot* [43]. Figures were created using *ggplot2* [44]. Analyzes were carried out for the two subgroups “mild to severe dementia” (MMSE ≥ 10) and “very severe dementia” (MMSE < 10) separately.

## Results

### Characteristics of the sample

Table 1 shows the sample characteristics. The sample consisted of 526 patients—344 people with mild to severe dementia, and 182 with very severe dementia. 60.6% of the participants were female. The mean age was 80.5 years and the average length of hospital stay was about 9.4 days. These characteristics were similar for both sub-groups (mild to severe and very severe dementia).

**Table 1:** Characteristics of the sample, shown are proportions of sample (%), or mean and standard deviation (in parenthesis)

Characteristic	Mild to severe dementia (n = 344)	Very severe dementia (n = 182)	Total (n = 526)	*p* value of difference
Proportion female, %	59.3	63.2	60.6	0.439^a^
Mean age (SD)	81.5 (9.5)	78.7 (12.1)	80.5 (10.6)	0.007^b^
Mean barthel-index (SD)	45.9 (28.5)	19.4 (24.4)	36.7 (29.9)	< 0.001^c^
Mean PAS-score (SD)	2.9 (3.1)	4.1 (3.3)	3.3 (3.2)	< 0.001^c^
Mean CCI (SD)	2.8 (1.6)	2.7 (1.6)	2.8 (1.6)	0.292^c^
Mean length of stay, in days (SD)	9.2 (5.4)	9.7 (7.8)	9.4 (6.3)	0.732^c^
Mean QUALIDEM total score (SD)	51.2 (16.0)	40.1 (16.5)	47.3 (17.0)	< 0.001^b^

Barthel-Index: 0–100 (higher = better functioning); QUALIDEM: 0–100 (higher = better QoL)

^a^χ^2^-test

^b^t-test

^c^Mann–Whitney-U test

**Fig. 1:**
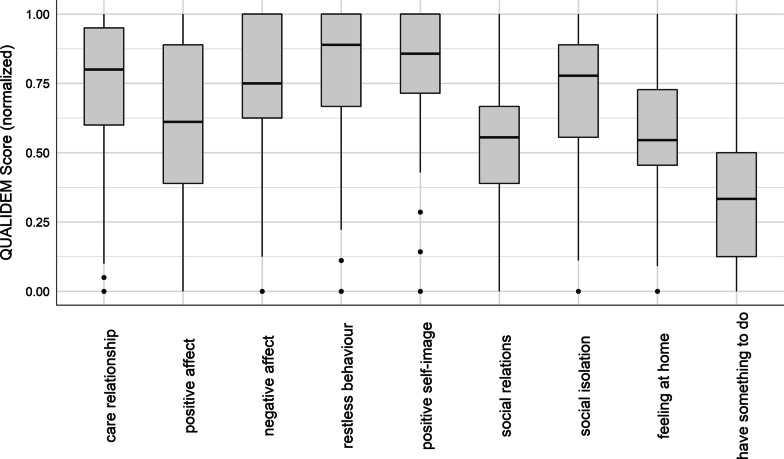
Distribution of QUALIDEM Scores from each subscale for patients with mild to severe dementia (n = 344)

**Fig. 2:**
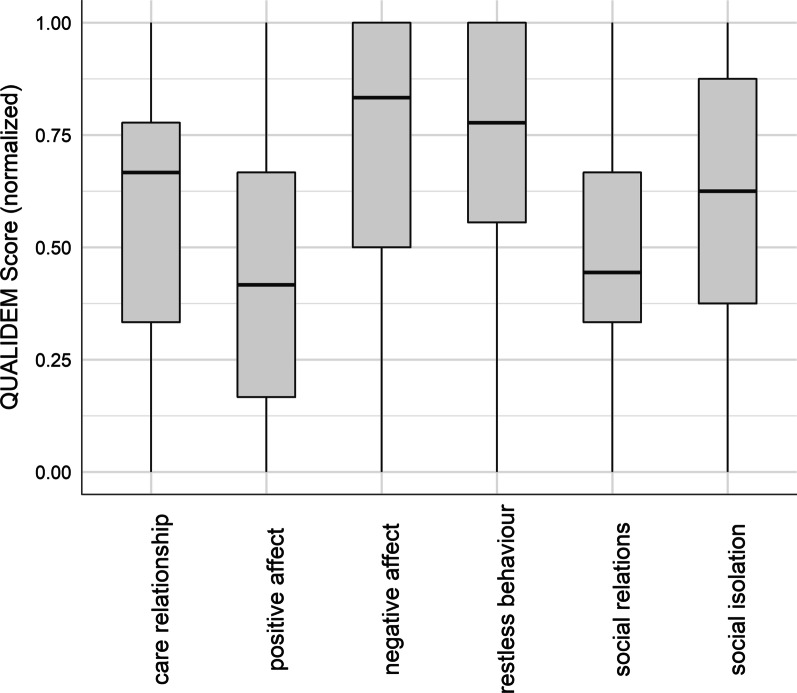
Distribution of QUALIDEM Scores from each subscale for patients with very severe dementia (n = 182)

The average Barthel-Index in the sample was 36.7, but comparably higher for people with mild to severe dementia (45.9) as opposed to those people with very severe dementia (19.4). According to the QoL, people with mild to severe dementia had a mean QUALIDEM-score of 51.2, while the group of people with severe dementia had a mean score of 40.1. To complete the sample description, we provided the mean values and their SD for each QUALIDEM subscale in Table 2. However, these are not directly comparable due to different numbers of items between the two groups and thereby different ranges for the subscales. Looking at the normalized scores of the QUALIDEM subscales for people with mild to severe dementia in Fig. 1, we found higher QoL for “care relationship”, “restless behavior”, “positive self-image” and “social isolation”, while especially the domain of “having something to do” is associated with the lowest QoL score. People with very severe dementia showed higher QoL scores for “negative affect” and “restless behavior”, while “positive affect” and “social relations” were those domains with the lowest QoL scores (Fig. 2).

**Table 2:** Sample characteristics of the QUALIDEM subscales, mean and standard deviation (in parenthesis)

Mean QUALIDEM subscale scores* (SD)	Mild to severe dementia (n = 344)	Very severe dementia (n = 182)
(A) Care relationship	16.0 (4.5)	5.1 (2.7)
(B) Positive affect	11.0 (5.1)	5.2 (3.6)
(c) Negative affect	6.9 (1.8)	4.5 (1.5)
(D) Restless tense behavior	7.2 (2.2)	6.5 (2.7)
(E) Positive self-image	7.5 (1.6)	NA
(F) Social relations	9.8 (3.6)	4.5 (2.1)
(G) Social Isolation	6.4 (2.2)	6.2 (2.3)
(H) Feeling at home	7.4 (2.1)	NA
(I) Having something to do	2.2 (1.8)	NA

^*^ Mean values are not directly comparable because number of items per subscale differ between patients with mild to severe and patients with very severe dementia

### Missing value analysis

Of the 37 QUALIDEM items for the group of people with mild to severe dementia, 612 out of 12,728 responses were missing (4.8%). For the people with very severe dementia, 350 out of 3276 responses of the 18 QUALIDEM items (10.7%) were missing.

### Item distribution

Table 3 shows the distribution of items of the QUALIDEM for people with mild to severe dementia. The distribution of items varies between the different subscales of the QUALIDEM. Eleven items out of six subscales (“care relationship”, “negative affect”, “restless tense behavior”, “positive self-image”, “social isolation” or “feeling at home”) showed a ceiling effect with a left-skewed distribution from “often” to “never”. In most cases, the response category for these items was “never” (from about 45% to 75%, except for the two items “cries” and “is rejected by other patients”, which have a proportion of 35.8% and 37.8%, respectively). 11 items show ceiling effects, while two items show floor effects. Those subscales where at least half of the items have ceiling or floor effects are “negative affect”, “positive self-image” and “feeling at home”. The items of the subscale “positive affect” showed a similar distribution with a peak at the response category “rarely”, so the ceiling effect was less evident. The other scales showed no consistent pattern across items.

**Table 3:** Item distribution (range 0–3) of nine QUALIDEM subscales for people with mild to severe dementia, including missing values and mean/SD (n = 344)

Item Nr	Subscale (Item)	0	1	2	3	Missing values	Item difficulty
**A**	**Care relationship**						
4	Rejects help from nursing assistants^a^	20 (5.8%)	40 (11.6%)	48 (14.0%)	217 (63.1%)	19 (5.5%)	0.81
7	Is angry^a^	33 (9.6%)	70 (20.3%)	82 (23.8%)	157 (45.6%)	2 (0.6%)	0.69
14	Has conflicts with nursing assistants^a^	29 (8.4%)	66 (19.2%)	76 (22.1%)	170 (49.4%)	3 (0.9%)	0.71
17	Accuses others^a^	26 (7.6%)	55 (16.0%)	61 (17.7%)	183 (53.2%)	19 (5.5%)	0.74
24	Appreciates help he or she receives^b^	29 (8.4%)	57 (16.6%)	90 (26.2%)	162 (47.1%)	6 (1.7%)	0.71
31	Accepts help^b^	11 (3.2%)	33 (9.6%)	90 (26.2%)	194 (56.4%)	16 (4.7%)	0.81
33	Criticizes the daily routine^a^	16 (4.7%)	38 (11.0%)	60 (17.4%)	225 (65.4%)	5 (1.5%)	0.82
**B**	**Positive affect**						
1	Is cheerful^b^	52 (15.1%)	78 (22.7%)	102 (29.7%)	98 (28.5%)	14 (4.1%)	0.58
5	Radiates satisfaction^b^	37 (10.8%)	72 (20.9%)	124 (36.0%)	105 (30.5%)	6 (1.7%)	0.63
8	Is capable of enjoying things in daily life^b^	25 (7.3%)	81 (23.5%)	134 (39.0%)	100 (29.1%)	4 (1.2%)	0.64
10	Is in a good mood^b^	39 (11.3%)	84 (24.4%)	124 (36.0%)	94 (27.3%)	3 (0.9%)	0.60
21	Has a smile around the mouth^b^	38 (11.0%)	85 (24.7%)	109 (31.7%)	98 (28.5%)	14 (4.1%)	0.60
40	Mood can be influenced in positive sense^b^	30 (8.7%)	97 (28.2%)	105 (30.5%)	109 (31.7%)	3 (0.9%)	0.62
**C**	**Negative affect**						
6	Makes an anxious impression^a^	16 (4.7%)	16 (4.7%)	40 (11.6%)	255 (74.1%)	17 (4.9%)	0.88
11	Is sad^a^	8 (2.3%)	18 (5.2%)	50 (14.5%)	248 (72.1%)	20 (5.8%)	0.89
23	Cries^a^	82 (23.8%)	69 (20.1%)	67 (19.5%)	123 (35.8%)	3 (0.9%)	0.56
**D**	**Restless tense behavior**						
2	Makes restless movements^a^	23 (6.7%)	50 (14.5%)	69 (20.1%)	196 (57.0%)	6 (1.7%)	0.77
19	Is restless^a^	13 (3.8%)	20 (5.8%)	51 (14.8%)	258 (75.0%)	2 (0.6%)	0.87
22	Has tense body language^a^	27 (7.8%)	44 (12.8%)	76 (22.1%)	182 (52.9%)	15 (4.4%)	0.75
**E**	**Positive self-image**						
27	Indicates he or she would like more help^a^	7 (2.0%)	21 (6.1%)	55 (16.0%)	243 (70.6%)	18 (5.2%)	0.88
35	Indicates not being able to do anything^a^	13 (3.8%)	50 (14.5%)	90 (26.2%)	173 (50.3%)	18 (5.2%)	0.77
37	Indicates feeling worthless^a^	19 (5.5%)	23 (6.7%)	27 (7.8%)	251 (73.0%)	24 (7.0%)	0.86
**F**	**Social relations**						
3	Has contact with other patients^b^	68 (19.8%)	95 (27.6%)	91 (26.5%)	68 (19.8%)	22 (6.4%)	0.50
12	Responds positively when approached^b^	6 (1.7%)	39 (11.3%)	110 (32.0%)	185 (53.8%)	4 (1.2%)	0.80
18	Takes care of other patients^b^	233 (67.7%)	34 (9.9%)	13 (3.8%)	16 (4.7%)	48 (14.0%)	0.12
25	Cuts himself/herself off from environment^a^	30 (8.7%)	59 (17.2%)	60 (17.4%)	179 (52.0%)	16 (4.7%)	0.73
29	Is on friendly terms with one or more patients^b^	135 (39.2%)	60 (17.4%)	65 (18.9%)	56 (16.3%)	28 (8.1%)	0.38
34	Feels at ease in the company of others^a^	37 (10.8%)	45 (13.1%)	32 (9.3%)	228 (66.3%)	2 (0.6%)	0.77
**G**	**Social Isolation**						
16	Is rejected by other patients^a^	81 (23.5%)	53 (15.4%)	76 (22.1%)	130 (37.8%)	4 (1.2%)	0.58
20	Openly rejects contact with others^a^	17 (4.9%)	23 (6.7%)	48 (14.0%)	241 (70.1%)	15 (4.4%)	0.85
32	Calls out^a^	39 (11.3%)	66 (19.2%)	64 (18.6%)	173 (50.3%)	2 (0.6%)	0.69
**H**	**Feeling at home**						
13	Indicates that he or she is bored^a^	19 (5.5%)	26 (7.6%)	45 (13.1%)	235 (68.3%)	19 (5.5%)	0.84
28	Indicates feeling locked up^a^	2 (0.6%)	20 (5.8%)	55 (16.0%)	237 (68.9%)	30 (8.7%)	0.89
36	Feels at home on the ward^b^	222 (64.5%)	36 (10.5%)	41 (11.9%)	20 (5.8%)	25 (7.3%)	0.19
39	Wants to get off the ward^a^	75 (21.8%)	78 (22.7%)	51 (14.8%)	120 (34.9%)	20 (5.8%)	0.59
**I**	**Having something to do**						
26	Finds things to do without help from others^b^	87 (25.3%)	83 (24.1%)	93 (27.0%)	65 (18.9%)	16 (4.7%)	0.47
38	Enjoys helping with chores on the ward^b^	145 (42.2%)	31 (9.0%)	28 (8.1%)	16 (4.7%)	124 (36.0%)	0.20

^a^CODING of items is 0 = often, 1 = sometimes, 2 = rarely, 3 = never

^b^CODING of items is 0 = never, 1 = rarely, 2 = sometimes, 3 = often

The distributions of the QUALIDEM items for people with very severe dementia (Table 4) show comparable patterns as in Table 3, however, with a less pronounced proportion of the response category “never”. Only two items show ceiling effects (“makes an anxious impression” and “openly rejects contact with others”). We found no floor effects in the six subscales of the QUALIDEM items for people with very severe dementia.

**Table 4:** Item distribution (range 0–3) of six QUALIDEM subscales for people with very severe dementia, including missing values and mean/SD (n = 182)

Item Nr	Subscale (Item)	0	1	2	3	Missing values	Item difficulty
**A**	**Care relationship**						
7	Is angry^a^	26 (14.3%)	29 (15.9%)	41 (22.5%)	80 (44.0%)	6 (3.3%)	0.66
14	Has conflicts with nursing assistants^a^	30 (16.5%)	42 (23.1%)	33 (18.1%)	71 (39.0%)	6 (3.3%)	0.61
31	Accepts help^b^	63 (34.6%)	43 (23.6%)	37 (20.3%)	32 (17.6%)	7 (3.8%)	0.41
**B**	**Positive affect**						
5	Radiates satisfaction^b^	39 (21.4%)	57 (31.3%)	50 (27.5%)	30 (16.5%)	6 (3.3%)	0.47
8	Is capable of enjoying things in daily life^b^	48 (26.4%)	49 (26.9%)	61 (33.5%)	18 (9.9%)	6 (3.3%)	0.43
21	Has a smile around the mouth^b^	53 (29.1%)	46 (25.3%)	48 (26.4%)	29 (15.9%)	6 (3.3%)	0.43
40	Mood can be influenced in positive sense^b^	50 (27.5%)	49 (26.9%)	51 (28.0%)	25 (13.7%)	7 (3.8%)	0.43
**C**	**Negative affect**						
6	Makes an anxious impression^a^	4 (2.2%)	4 (2.2%)	10 (5.5%)	101 (55.5%)	63 (34.6%)	0.92
23	Cries^a^	55 (30.2%)	30 (16.5%)	17 (9.3%)	72 (39.6%)	8 (4.4%)	0.54
**D**	**Restless tense behavior**						
2	Makes restless movements^a^	26 (14.3%)	38 (20.9%)	33 (18.1%)	79 (43.4%)	6 (3.3%)	0.65
19	Is restless^a^	18 (9.9%)	16 (8.8%)	21 (11.5%)	123 (67.6%)	4 (2.2%)	0.80
22	Has tense body language^a^	11 (6.0%)	26 (14.3%)	21 (11.5%)	62 (34.1%)	62 (34.1%)	0.71
**F**	**Social relations**						
3	Has contact with other patients^b^	98 (53.8%)	36 (19.8%)	22 (12.1%)	6 (3.3%)	20 (11.0%)	0.20
12	Responds positively when approached^b^	23 (12.6%)	38 (20.9%)	67 (36.8%)	50 (27.5%)	4 (2.2%)	0.60
25	Cuts himself/herself off from environment^a^	22 (12.1%)	29 (15.9%)	15 (8.2%)	55 (30.2%)	61 (33.5%)	0.62
**G**	**Social Isolation**						
16	Is rejected by other patients^a^	57 (31.3%)	22 (12.1%)	23 (12.6%)	73 (40.1%)	7 (3.8%)	0.55
20	Openly rejects contact with others^a^	9 (4.9%)	7 (3.8%)	13 (7.1%)	90 (49.5%)	63 (34.6%)	0.85
32	Calls out^a^	49 (26.9%)	36 (19.8%)	17 (9.3%)	72 (39.6%)	8 (4.4%)	0.55

### Floor and ceiling effects for QUALIDEM subscales

Six out of nine subscales ("care relationship”, “positive affect”, “negative affect”, “restless tense behavior”, “positive self-image” and “social isolation”) showed significant ceiling effects for the group of patients with mild to severe dementia. Significant floor effects for this group were found in one subscale (“having something to do”). The total score of the QUALIDEM showed no floor nor ceiling effects. For patients with very severe dementia, three out of six subscales showed significant ceiling effects (“negative affect” and “social isolation”), while “positive affect” was the only subscale with a significant floor effect (see Table 5).

**Table 5:** Floor and ceiling effects for QUALIDEM subscales and total score, for patients with mild to severe dementia (n = 344) and patients with very severe dementia (n = 182)

Item Nr	Subscale (Item)	Mild to severe dementia		Very severe dementia	
		Flooring (%)	Ceiling (%)	Flooring (%)	Ceiling (%)
A	Care relationship	0.6	34.9*	6.6	9.9
B	Positive affect	4.4	20.9*	19.8*	8.2
C	Negative affect	0.0	27.0*	1.1	26.9*
D	Restless tense behavior	0.9	39.8*	2.7	24.2*
E	Positive self-image	0.0	36.3*	–	–
F	Social relations	0.6	3.8	2.7	1.1
G	Social isolation	0.9	21.8*	0.5	17.0*
H	Feeling at home	0.3	5.8	–	–
I	Having something to do	20.9*	2.9	–	–
Total	QUALIDEM-score	0.0	5.5	0.0	4.4

^*^Effects were considered significant if the proportion of floor or ceiling effects exceeded 15%

### Known-group validity

Table 6 shows the results for the known-group validity. For patients with mild to severe dementia, all a priori defined hypotheses were accepted, indicating a high validity of the QUALIDEM score for the five defined groups. Medium to large effects were found for differences between the distinct groups “lower/higher agitation and challenging behavior” and “lower/higher comorbidities”. For people with very sever dementia, only the hypothesis that patients with higher comorbidities had a lower QoL was rejected. Differences between the distinct groups “lower/higher agitation and challenging behavior” and “lower/higher comorbidities” were considered as large effects.

**Table 6:** Known-group validity for the QUALIDEM score for five distinct groups, by patients with mild to severe dementia (n = 344) and patients with very severe dementia (n = 182)

Hypothesis	Mild to severe dementia					
	Cohen’s d	p*	Decision	Cohen’s d	p*	Decision
No significant difference in QoL between younger and older elderly patients	0.11	0.319^a^	Accept	0.15	0.346^a^	Accept
No significant difference in QoL between female and male patients	0.10	0.403^a^	accept	0.14	0.388^a^	Accept
QoL is significantly lower for patients with higher functional limitations	0.51	< 0.001^b^	Accept	0.90	< 0.001^b^	Accept
QoL is significantly lower for patients with higher agitation and challenging behavior	1.17	< 0.001^b^	Accept	1.35	< 0.001^b^	Accept
QoL is significantly lower for patients with a higher number of comorbidities	0.21	0.029^b^	Accept	0.22	0.072^b^	Reject

^*^p values for t-test, indicating statistically significant differences of QUALIDEM scores between distinct groups

^a^TWO-sided t-test

^b^ONE-sided t-test

### Scalability

Table 7 shows the results of the MSA from the QUALIDEM for patients with mild to severe dementia. Three of the nine subscales show strong scalability (“positive affect”, H = 0.77; “restless tense behavior”, H = 0.55; “having something to do”, H = 0.56). The subscales “care relationship” and “social relations” have moderate scalability (H = 0.43 and H = 0.47 respectively). Most of their items were also scalable, with exception of “rejects help from nursing assistants” (H = 0.24) and “feels at ease in the company of others” (H = 0.28). “Negative affect” (H = 0.31) and “social isolation” (H = 0.32) show weak scalability. The items “is sad” (H = 0.26) and “is rejected by other patients” (H = 0.28) are not scalable. The subscales “positive self-image” (H = 0.17) and “feeling at home” (H = 0.16) were not scalable.

**Table 7:** Scalability and internal consistency from nine QUALIDEM subscales for people with mild to severe dementia (n = 344), 5% of all items have missing values (612 out of 12,728 data points from items are missing)

Item Nr	Subscale (Item)	Qualidem David			Dichter et al. [30] (Total)			Arons et al. [31]	
		Scale-H (Item H_i_)	ρ	Cronbach’s α	Scale-H (Item H_i_)	ρ	Cronbach’s α	Scale-H (Item H_i_)	*ρ*
**A**	**Care relationship**	**0.43**	**0.82**	**0.82**	**0.42**	**0.81**	**0.81**	**0.45**	**0.80**
4	Rejects help from nursing assistants	0.24			0.48			0.47	
7	Is angry	0.51			0.49			0.51	
14	Has conflicts with nursing assistants	0.51			0.52			0.56	
17	Accuses others	0.33			0.32			0.41	
24	Appreciates help he or she receives	0.47			0.39			0.30	
31	Accepts help	0.42			0.43			0.36	
33	Criticizes the daily routine	0.51			0.31			0.44	
**B**	**Positive affect**	**0.77**	**0.95**	**0.94**	**0.65**	**0.91**	**0.90**	**0.65**	**0.90**
1	Is cheerful	0.80			0.67			0.66	
5	Radiates satisfaction	0.78			0.69			0.67	
8	Is capable of enjoying things in daily life	0.73			0.62			0.64	
10	Is in a good mood	0.81			0.71			0.72	
21	Has a smile around the mouth	0.78			0.66			0.66	
40	Mood can be influenced in positive sense	0.70			0.52			0.54	
**C**	**Negative affect**	**0.31**	**0.48**	**0.45**	**0.53**	**0.73**	**0.72**	**0.62**	**0.80**
6	Makes an anxious impression	0.33			0.49			0.55	
11	Is sad	0.26			0.59			0.65	
23	Cries	0.33			0.52			0.65	
**D**	**Restless tense behavior**	**0.55**	**0.76**	**0.74**	**0.45**	**0.69**	**0.68**	**0.36**	**0.61**
2	Makes restless movements	0.53			0.51			0.41	
19	Is restless	0.53			0.51			0.35	
22	Has tense body language	0.58			0.32			0.32	
**E**	**Positive self-image**	**0.17**	**0.35**	**0.34**	**0.42**	**0.67**	**0.67**	**0.64**	**0.83**
27	Indicates he or she would like more help	0.12			0.36			0.64	
35	Indicates not being able to do anything	0.17			0.50			0.62	
37	Indicates feeling worthless	0.22			0.41			0.66	
**F**	**Social relations**	**0.47**	**0.79**	**0.76**	**0.43**	**0.77**	**0.73**	**0.30**	**0.65**
3	Has contact with other patients	0.47			0.47			0.40	
12	Responds positively when approached	0.46			0.44			0.34	
18	Takes care of other patients	0.67			0.42			0.24	
25	Cuts himself/herself off from environment	0.45			0.33			0.15	
29	Is on friendly terms with one or more patients	0.55			0.45			0.37	
34	Feels at ease in the company of others	0.28			0.48			0.38	
**G**	**Social Isolation**	**0.32**	**0.52**	**0.52**	**0.28**	**0.53**	**0.52**	**0.44**	**0.69**
16	Is rejected by other patients	0.28			0.35			0.46	
20	Openly rejects contact with others	0.36			0.29			0.45	
32	Calls out	0.32			0.21			0.39	
**H**	**Feeling at home**	**0.16**	**0.36**	**0.29**	**0.31**	**0.62**	**0.61**	**0.51**	**0.77**
13	Indicates that he or she is bored	0.09			0.26			0.52	
28	Indicates feeling locked up	0.11			0.34			0.62	
36	Feels at home on the ward	0.12			0.30			0.20	
39	Wants to get off the ward	0.28			0.34			0.58	
**I**	**Having something to do**	**0.56**	**0.69**	**0.64**	**0.18**	**0.23**	**0.24**	**0.39**	**0.53**
26	Finds things to do without help from others	0.56			0.18			0.39	
38	Enjoys helping with chores on the ward	0.56			0.18			0.39	

Item numbers in tables correspond to those in Dichter et al. and Arons et al. to make comparison easier

Bold values refer to the subscale

The MSA for the group of people with very severe dementia is shown in Table 8. All six subscales were scalable (0.34 ≤ H ≤ 0.71). The scalability could be considered as weak for “social relations”, moderate for “social isolation” and strong for the other remaining four subscales.

**Table 8:** Scalability and internal consistency from six QUALIDEM subscales for people with very severe dementia (n = 182), 11% of all items have missing values (350 out of 3276 data points from items are missing)

Item Nr.	Subscale (Item)	Qualidem David			Dichter et al. [30] (Total)			Arons et al. [31] (2017)	
		Scale-H (Item H_i_)	ρ	Cronbach’s α	Scale-H (Item H_i_)	ρ	Cronbach’s α	Scale-H (Item H_i_)	ρ
**A**	**Care relationship**	**0.50**	**0.74**	**0.70**	**0.47**	**0.73**	**0.67**	**0.43**	**0.79**
7	Is angry	0.55			0.54			0.53	
14	Has conflicts with nursing assistants	0.58			0.54			0.56	
31	Accepts help	0.36			0.35			0.41	
**B**	**Positive affect**	**0.71**	**0.91**	**0.90**	**0.65**	**0.86**	**0.85**	**0.65**	**0.90**
5	Radiates satisfaction	0.72			0.70			0.66	
8	Is capable of enjoying things in daily life	0.71			0.64			0.64	
21	Has a smile around the mouth	0.75			0.65			0.68	
40	Mood can be influenced in positive sense	0.67			0.59			0.61	
**C**	**Negative affect**	**0.65**	**0.69**	**0.62**	**0.36**	**0.50**	**0.47**	**0.61**	**0.77**
6	Makes an anxious impression	0.64			0.36			0.51	
23	Cries	0.64			0.36			0.65	
**D**	**Restless tense behavior**	**0.65**	**0.83**	**0.80**	**0.37**	**0.59**	**0.62**	**0.38**	**0.63**
2	Makes restless movements	0.60			0.47			0.48	
19	Is restless	0.61			0.45			0.43	
22	Has tense body language	0.72			0.18			0.24	
**F**	**Social relations**	**0.34**	**0.55**	**0.53**	**0.33**	**0.53**	**0.52**	**0.34**	**0.60**
3	Has contact with other patients	0.27			0.36			0.38	
12	Responds positively when approached	0.42			0.34			0.43	
25	Cuts himself/herself off from environment	0.32			0.30			0.21	
**G**	**Social isolation**	**0.45**	**0.65**	**0.66**	**0.20**	**0.42**	**0.41**	**0.41**	**0.66**
16	Is rejected by other patients	0.39			0.25			0.46	
20	Openly rejects contact with others	0.56			0.21			0.39	
32	Calls out	0.42			0.13			0.37	

Bold values refer to the subscale

### Internal consistency

From the nine subscales of the QUALIDEM for people with mild to severe dementia, only five showed acceptable to excellent internal consistencies varying from ρ = 0.69 to 0.95 (“care relationship”, “positive affect”, “restless tense behavior”, “social relations” and “having something to do”, see Table 7). Five out of six subscales from the QUALIDEM for people with very severe dementia showed at least acceptable internal consistencies (ρ = 0.65–0.91, Table 8). Only “social relations” had an insufficient reliability (ρ = 0.55).

## Discussion

The aim of the current study was to investigate whether the item distribution, scalability and internal consistency of the dementia-specific QUALIDEM instrument can be replicated in a hospital context. As a reference for comparison, we chose one study from Dichter et al. [45] and one from Arons et al. [46], which represent recent works on analyzing the item distribution and testing the scalability and internal consistency of the QUALIDEM in nursing home settings.

### Item distribution

The investigation of the item distribution of the QUALIDEM demonstrated a moderately balanced distribution of the four response options. Twenty-six out of 37 items for people with mild to severe dementia showed an acceptable item difficulty, and only two out of 18 items for people with very severe dementia showed a ceiling effect. The proportion of missing values varies from 0.6 to 36.0% and is not always in an acceptable range (< 10%); this particularly holds true for the items in the “social relations” dimension. Here the proportion of missing values was high due to the frequent use of the failure rating category “not applicable”. One reason for these results might be a missing cross-cultural adaption of the QUALIDEM measurement for the German context and in particular for German hospital settings.

These descriptive findings are widely in line with previous results. Yet, Arons et al. [46], for example, reported that with one exception (item “feels at home on the ward”) all other items had less than 1% missing values. A recent study by Dichter et al. [47] showed fewer ceiling effects, however, the German-language QUALIDEM version 2.0 was used here, which offers a total of seven assessment options to choose from (“never”, “very rarely”, “rarely”, “sometimes”, “often”, “frequently” and “very frequently”). In the present study, the original German version 1.0 of the QUALIDEM was used with only four assessment options. Hence, the small number of rating options could be the reason for the high number of ceiling effects (and lower internal consistency).

It is also noticeable that almost all item raw scores in seven subscales for people with mild to severe dementia, but no items in two subscales (”positive affect”, “having something to do”) are left-skewed in distribution. The most obvious right-skewness in one dimension appears in item 18 (“takes care of other patients”). Here, unlike in other items, negative assessments by study nurses are dominant. Researchers must consider the challenges inherent in rating before determining the QoL outcome and adapt their methodological approaches accordingly.

### Floor and ceiling effects for QUALIDEM subscales and total score

Regarding the QUALIDEM subscales, we found floor or ceiling effects for six (out of nine) subscales for patients with mild to severe dementia, and three (out of six) subscales for patients with very severe dementia. No ceiling or floor effects were found for the QUALIDEM total scores in both groups. Although ceiling and floor effects can be a critical issue for outcomes such as QoL, we consider them being less of a concern for the QUALIDEM. To a certain extent, the small number of items per subscale, which affects a scale’s discrimination, can explain the rather high proportions of floor or ceiling effects. However, it remains unclear whether the effects we found were only statistically or also clinically significant. This suggests using the QUALIDEM total score or getting a differentiated picture by looking at all subscales and not at isolated subscales only.

### Known-group validity

The known-group validity is a construct validity that can be used to test whether a scale is able differentiate between distinct groups where differences were to be expected a priori. We derived five hypotheses based on former research about predictors of QoL for PwD [25, 26]. For patients with mild to severe dementia, we found evidence for all hypotheses we put forward. Only one hypothesis was rejected for the group of patients with very severe dementia. Where we expected no differences between distinct groups, effect sizes were also very small. We found medium to large effect sizes for those distinct groups where differences in the QUALIDEM score were expected. Only the distinction between PwD with lower versus higher number of comorbidities showed small effect sizes. This suggests that the QUALIDEM instrument was able to detect valid differences between patients with different characteristics.

### Scalability

The subscales “care relationship” and “social relations” have moderate scalability, but still scoring good or slightly better than the same subscales in the previous studies [45, 46]. The subscale “care relationship” might be improved by omitting the items “rejects help from nursing assistants” (item 4) and “accuses others” (item 17). Regarding the subscale “social relations”, the same holds true for the item “feels at ease in the company of others” (item 34). Especially the item “rejects help from nursing assistants” had a higher scalability in both studies by Dichter et al. [45] and Arons et al. [46]. This indicates that a specific adaptation of the QUALIDEM for hospital settings seems reasonable.

“Negative affect” and “social isolation” show weak scalability. While the result for “social isolation” is at least comparable to Dichter et al. [45], “negative affect” has a remarkably lower scalability compared to the other study. These results are less surprising, given that limitations according to either weak or inconsistent scalability of these two subscales have also been recognized by the authors of the QUALIDEM instrument [11]. One explanation might be difficulties according to the interrater reliability. Personal interviews with people using the QUALIDEM revealed that items like “cries” or “is sad” are interpreted in very different ways, which seems to make those items prone to subjectively biased perceptions of patients’ moods.

The subscales “positive self-image” and “feeling at home” were not scalable. We assume that both the hospital setting as well as the shorter observation period—as compared to nursing homes—might explain these results for the items of these two subscales. Looking at single items, the item “wants to get off the ward” (item 39) has a comparably higher scalability than the remaining items of the subscale “feeling at home”, which is reasonable in a hospital context. The distributions of responses to this item has a rather uniform shape. This implies that there is a notable number of PwD, who want to get off the ward. When it comes to revising the QUALIDEM for a hospital context, this item should still be considered in order to adequately measure QoL.

Within the group of patients with very severe dementia, we found strong scalability for “care relationship”, “positive affect”, “negative affect” and “restless tense behavior”. The differences in scalability between the group of mild to severe dementia and very severe dementia can partly be explained by the reduced number of items for some subscales in the latter group. Low scalable items like “rejects help from nursing assistants” (item 4) or “accuses others” (item 17) were removed from the subscale “care relationship” in the reduced QUALIDEM version for patients with very severe dementia. However, the items of “negative affect” have a much higher scalability for patient with very severe dementia as compared to the group with mild to severe dementia.

### Internal consistency

The internal consistency results only partially correspond with results of the reference studies by Dichter et al. [45] and Arons et al. [46]. For patients with mild to severe dementia, the subscales "care relationship", "positive affect", “restless, tense behavior”, “social relations” and “having something to do” showed similar acceptable to excellent internal consistencies. Comparatively, there was significantly less homogeneity for the subscale “negative affect”, "positive self-image" and "feeling at home". In accordance with both studies, an insufficient level of internal consistency was determined for the subscale “social isolation”, while better characteristics (rho, alpha) were only found for “having something to do”.

The QUALIDEM subscales for people with a very severe dementia showed similar results as in the previous studies [45, 46]. For the subscales “care relationship”, “positive affect”, “negative affect”, “restless, tense behavior” and “social isolation” a good homogeneity could be determined—even better values in three subscales. Comparably, the subscale “social relations” showed a similarly poor internal consistency. One reason for lower Cronbach's alpha values could be rather small number of items in the subscales. This is typical for Cronbach’s alpha values. They increase as the number of items increases [48].

Our main finding suggests that for most of the subscales, especially for the group of people with very severe dementia, the results of the internal consistency analysis as well as the MSA were at least as good as in the two reference studies, and sometimes even better. Nevertheless, for all subscales, 50% of the proxy participants reached a score of 50 or higher, regardless of dementia severity. This result raises the question of QUALIDEM’s sensitivity for change, which has not been assessed. Information on responsiveness is scarce in general, which highlights the need for research on this topic. To use QoL as an outcome in intervention studies, evidence of the QUALIDEM’s sensitivity for change is required.

### Strength and limitations

The article is based on the first study using data from inpatient care to analyze psychometrics of the dementia-specific QUALIDEM instrument in Germany. There are, however, a number of limitations. Compared to other studies using the QUALIDEM, we had a slightly higher proportion of missing values in some items, but tackling this issue with imputation techniques is feasible. Missing values in psychometric testing are not a problem per se, but may result in biased reliability scores [49]. Therefore, we have compared results using two different imputations techniques and the per-protocol data (i.e., no imputation of missing values, see Additional file 1), which suggests that the impact of missing values in our study is negligible. Individual results relating item difficulty may be enhanced by using German-language QUALIDEM version 2.0, which did not yet exist at the time the data was collected in the DAVID project. Furthermore, reliability scores (ρ, Cronbach’s α) were problematic for scales with less than 10 items. This problem was already identified by the authors of the QUALIDEM [11], which led to the development of the revised second version of this assessment instrument. Unfortunately, it was not possible to measure the interrater reliability in the DAVID project. Thus, we could not clearly identify the causes for the low scalability scores of some subscales. Another limitation of the study relates to the hypothesizing. During preparatory work for the study, it was only possible to fall back on preliminary empirical findings in the context of formation of hypotheses, which were difficult to interpret due to the use of different assessment instruments. Despite these limitations, one of the first applications in hospital context is arguably a strength of this study, providing evidence that the QUALIDEM is a useful tool to measure QoL of PwD in hospitals.

## Conclusions

Despite the limitations mentioned above (most are general difficulties in measuring QoL) the instrument’s psychometric properties justify its use in the context of hospital research. In comparison with a previous evaluation of the scalability and reliability of the QUALIDEM in a long-term care setting, the application in a hospital setting leads to very similar, acceptable results for people with mild to severe dementia. For people with very severe dementia, our results suggest that the QUALIDEM instrument seems to fit even better in a hospital context as compared to long-term care settings. However, this result should be taken with a grain of salt, because the lower sample size and higher proportion of missing values only allow for limited evidence of this conclusion. Results suggest either a revision of unsatisfactory items or a general reduction to six or seven subscales for all PwD. In addition, an investigation of the inter-rater reliability of the QUALIDEM is recommended because the qualification of the nurses and the length of stay of the patients in the hospital differ from the previous investigations of the inter-rater reliability of QUALIDEM in the nursing home.

## Supplementary Information

**Additional file 1: Table A1.** Scalability and internal consistency from nine QUALIDEM subscales for people with mild to severe dementia for two different imputation methods and per-protocol data.

## Data Availability

The dataset supporting the conclusions of this article as well as the R source code to reproduce the results are available in the OSF repository [50] at https://osf.io/vunmf/.
