# Supplementary material for: Item distribution, scalability and internal consistency of the QUALIDEM quality of life assessment for patients with dementia in acute hospital settings
Source: Health Qual Life Outcomes. 2023 Jan 31;21:12. doi: 10.1186/s12955-023-02094-1 (PMC9887877; doi:10.1186/s12955-023-02094-1)
Supplement: Supplementary file 1 — Additional file 1: Table A1. Scalability and internal consistency from nine QUALIDEM subscales for people with mild to severe dementia for two different imputation methods and per-protocol data. [file 12955_2023_2094_MOESM1_ESM.docx]

Table A1: Scalability and internal consistency from nine QUALIDEM subscales for people with mild to severe dementia (n=344), 5% of all items have missing values (612 out of 12.728 data points from items are missing), for two different imputation methods and per-protocol data (no missing value imputation)

|  |  | QUALIDEM DAVID – Two-Way-Imputation | QUALIDEM DAVID – Multiple Imputation by Chained Equation | QUALIDEM DAVID – Per protocol (no imputed data) |
| --- | --- | --- | --- | --- |
| Item Nr. | Subscale (Item) | Scale-H (Rho) | Scale-H (Rho) | Scale-H (Rho) |
| **A** | **Care Relationship** | **.43 (.82)** | **.41 (.81)** | **.42 (.81)** |
| 4. | Rejects help from nursing assistants | .24 | .20 | .21 |
| 7. | Is angry | .51 | .50 | .50 |
| 14. | Has conflicts with nursing assistants | .51 | .50 | .51 |
| 17. | Accuses others | .33 | .30 | .30 |
| 24. | Appreciates help he or she receives | .47 | .46 | .47 |
| 31. | Accepts help | .42 | .40 | .41 |
| 33. | Criticizes the daily routine | .51 | .49 | .50 |
| **B** | **Positive Affect** | **.77 (.95)** | **.76 (.94)** | **.77 (.95)** |
| 1. | Is cheerful | .80 | .79 | .80 |
| 5. | Radiates satisfaction | .78 | .77 | .78 |
| 8. | Is capable of enjoying things in daily life | .73 | .73 | .72 |
| 10. | Is in a good mood | .81 | .81 | .81 |
| 21. | Has a smile around the mouth | .78 | .78 | .78 |
| 40. | Mood can be influenced in positive sense | .70 | .70 | .70 |
| **C** | **Negative Affect** | **.31 (.48)** | **.20 (.37)** | **.22 (.38)** |
| 6. | Makes an anxious impression | .33 | .23 | .25 |
| 11. | Is sad | .26 | .17 | .16 |
| 23. | Cries | .33 | .21 | .25 |
| **D** | **Restless tense behavior** | **.55 (.76)** | **.55 (.75)** | **.55 (.75)** |
| 2. | Makes restless movements | .53 | .53 | .53 |
| 19. | Is restless | .53 | .53 | .55 |
| 22. | Has tense body language | .58 | .57 | .57 |
| **E** | **Positive self-image** | **.17 (.35)** | **.15 (.32)** | **.15 (.32)** |
| 27. | Indicates he or she would like more help | .12 | .10 | .09 |
| 35. | Indicates not being able to do anything | .17 | .16 | .16 |
| 37. | Indicates feeling worthless | .22 | .20 | .20 |
| **F** | **Social relations** | **.47 (.79)** | **.43 (.77)** | **.44 (.78)** |
| 3. | Has contact with other residents | .47 | .43 | .43 |
| 12. | Responds positively when approached | .46 | .45 | .45 |
| 18. | Takes care of other residents | .67 | .59 | .66 |
| 25. | Cuts himself/herself off from environment | .45 | .42 | .42 |
| 29. | Is on friendly terms with one or more residents | .55 | .52 | .52 |
| 34. | Feels at ease in the company of others | .28 | .26 | .25 |
| **G** | **Social Isolation** | **.32 (.52)** | **.30 (.50)** | **.30 (.50)** |
| 16. | Is rejected by other residents | .28 | .27 | .26 |
| 20. | Openly rejects contact with others | .36 | .33 | .33 |
| 32. | Calls out | .32 | .30 | .30 |
| **H** | **Feeling at home** | **.16 (.36)** | **.09 (.23)** | **.12 (.31)** |
| 13. | Indicates that he or she is bored | .09 | .05 | .06 |
| 28. | Indicates feeling locked up | .11 | .04 | .07 |
| 36. | Feels at home on the ward | .12 | .01 | .06 |
| 39. | Wants to get off the ward | .28 | .20 | .25 |
| **I** | **Having something to do** | **.56 (.69)** | **.42 (.54)** | **.52 (.61)** |
| 26. | Finds things to do without help from others | .56 | .42 | .52 |
| 38. | Enjoys helping with chores on the ward | .56 | .42 | .52 |

Item numbers in tables correspond to those in Dichter et al. and Arons et al. to make comparison easier.
